# Detection of multiple human enteropathogens in Norway rats (*Rattus norvegicus*) from an under-resourced neighborhood of Vancouver, British Columbia

**DOI:** 10.1371/journal.pntd.0011669

**Published:** 2023-10-16

**Authors:** Lisa K. F. Lee, Chelsea G. Himsworth, Kaylee A. Byers, Harveen K. Atwal, Gus Gabaldon, Gordon Ritchie, Christopher F. Lowe, Nancy Matic, Samuel Chorlton, Linda Hoang, Bruce K. Wobeser, Victor Leung

**Affiliations:** 1 British Columbia Regional Centre, Canadian Wildlife Health Cooperative, Abbotsford, British Columbia, Canada; 2 Department of Veterinary Pathology, Western College of Veterinary Medicine, Saskatoon, Saskatchewan, Canada; 3 School of Population and Public Health, University of British Columbia, Vancouver, British Columbia, Canada; 4 Abell Pest Control, Vancouver, British Columbia, Canada; 5 Department of Pathology and Laboratory Medicine, University of British Columbia, Vancouver, British Columbia, Canada; 6 Division of Medical Microbiology, Department of Pathology and Laboratory Medicine, Providence Health Care, Vancouver, British Columbia, Canada; 7 British Columbia Centre for Disease Control, Vancouver, British Columbia, Canada; 8 Division of Infectious Diseases, Department of Medicine, Providence Health Care, Vancouver, British Columbia, Canada; Wadsworth Center, UNITED STATES

## Abstract

Urban Norway rats (*Rattus norvegicus*) can carry various human pathogens, and may be involved in pathogen propagation and transmission to humans. From January 31–August 14, 2021, a community outbreak of *Shigella flexneri* serotype 2a occurred among unhoused or poorly housed people in the Downtown Eastside neighborhood of Vancouver, British Columbia, Canada. The source could not be identified; however, patients reported contact with rats, and previous studies indicated transmission of rat-associated zoonotic pathogens among the unhoused or poorly housed residents of this neighborhood. The study objective was to determine if rats trapped in the outbreak area were carriers of *Shigella* spp. and other zoonotic enteric pathogens. From March 23–April 9, 2021, 22 rats were lethally trapped within the outbreak area. Colonic content was analyzed using the BioFire FilmArray Gastrointestinal (multiplex PCR) panel for human enteropathogens, which detected: *Campylobacter* spp. (9/22), *Clostridioides difficile* (3/22), *Yersinia enterocolitica* (5/22), *Cryptosporidium* spp. (8/22), *Giardia duodenalis* (5/22), Rotavirus A (1/22), enteroaggressive *Escherichia coli* (2/22), enteropathogenic *E*. *coli* (10/22), and *Shigella* spp. or enteroinvasive *E*. *coli* (EIEC) (3/22). An *ipaH* PCR assay was used for targeted detection of *Shigella* spp./EIEC, with five rats positive. Two samples contained insertion sites unique to *S*. *flexneri* isolated from the human outbreak. This study highlights the potential for rats to carry a broad range of human pathogens, and their possible role in pathogen maintenance and/or transmission.

## Author summary

Wild city rats can carry infectious organisms including various bacteria, protozoa, and viruses that are capable of causing disease in humans. People living in low-income areas or unhoused may have increased contact with rats and therefore may be particularly vulnerable. However, little is known regarding the role that rats have in spreading these organisms during human outbreaks of disease. From January to August 2021, there was an outbreak of *Shigella flexneri*, a bacteria that causes gastrointestinal disease, among homeless or poorly housed people in the Downtown Eastside neighborhood of Vancouver, Canada. We hypothesized that rats may have been involved in propagating the outbreak, so we trapped rats within the outbreak area and compared *Shigella* spp. strains from the feces of these rats and human patients. We found that some Norway rats were carrying *Shigella* spp., with two samples showing relatedness to the human samples. Rats were often carrying more than one potential human pathogen in their feces. This study highlights the importance of managing rat populations to reduce the spread of diseases to humans, especially among low-income neighborhoods with marginalized, unhoused, or poorly housed individuals.

## Introduction

Norway rats (*Rattus norvegicus*) carry a variety of zoonotic pathogens (bacteria, viruses, and parasites that can be transmitted from rats to people) responsible for significant human morbidity and mortality [1]. These pathogens can be broadly classified into two groups: 1) pathogens for which rats are the natural reservoir, such as *Leptospira interrogans*, Seoul hantavirus, and *Rickettsia typhi* (i.e., rat-origin pathogens); and 2) pathogens that rats acquire from other species, including humans, which they might subsequently propagate, such as methicillin-resistant *Staphylococcus aureus*, *Clostridioides difficile* (formerly *Clostridium difficile*), and *Escherichia coli* [2] (i.e., human-origin pathogens).

For rat-origin pathogens, the rodent reservoir plays a clear role in the epidemiology of human outbreaks. However, there is limited evidence showing the involvement of rats in pathogen transmission/propagation during outbreaks of human-origin pathogens in people. From January 31 to August 14, 2021, a community outbreak of *Shigella flexneri* serotype 2a occurred among people who were unhoused or living poorly housed in single room occupancy hotels in the under-resourced Downtown Eastside (DTES) neighborhood of Vancouver, British Columbia, Canada. As the source could not be identified, this highlighted the question of whether rats may be involved in pathogen propagation. Patients had reported contact with rats, and previous studies indicated significant rat exposure among this population [3], as well as transmission of other rat-associated zoonotic pathogens [4]. There is little access to public sanitation facilities in the DTES [5]; therefore, human fecal contamination in alleys where rats are known to reside is common and presents a pathway for human-to-rat pathogen transmission. Rat-to-human pathogen transmission is also possible via rat fecal contamination of areas where people dwell. It was therefore hypothesized that rats could be contributing to outbreak propagation.

Rats are not widely considered a source of *Shigella* spp. for people; however, *Shigella* spp./enteroinvasive *E*. *coli* (EIEC) were previously detected in rat feces from New York, USA [6], Tehran, Iran [7], and North West Province, South Africa [8]. These studies were conducted in the absence of a human outbreak. The main objective of this study was to determine if rats within the outbreak area at the time of the outbreak were carrying *S*. *flexneri*. A secondary objective was to determine if those rats were carrying other human enteropathogens.

## Materials and methods

### Ethics statement

This study was approved by the University of British Columbia’s Animal Care Committee (A20-0212) and adhered to national guidelines set out by the Canadian Council on Animal Care.

### Outbreak context

A cluster of patients with *S*. *flexneri* infection was detected on February 20, 2021. The incidence of cases increased rapidly and a community outbreak was officially announced on February 26, 2021 and declared over on August 14, 2021. The outbreak affected residents in the DTES, a neighbourhood of Vancouver, and an area where many marginalized people reside [5]. This area is noted for poverty, and a high number of unhoused or poorly housed people. Single room occupancy hotels are common in the DTES, and feature small rooms with shared bathrooms and kitchens. Since 2018, there has been more widespread housing displacement from rising rents, ultimately leading to the city’s highest homelessness rates since 2005. A summary of this outbreak has not been published, but there were 65 confirmed cases of *S*. *flexneri* serotype 2a and significantly more probable cases.

### Site selection and sample collection

**Fig 1:**
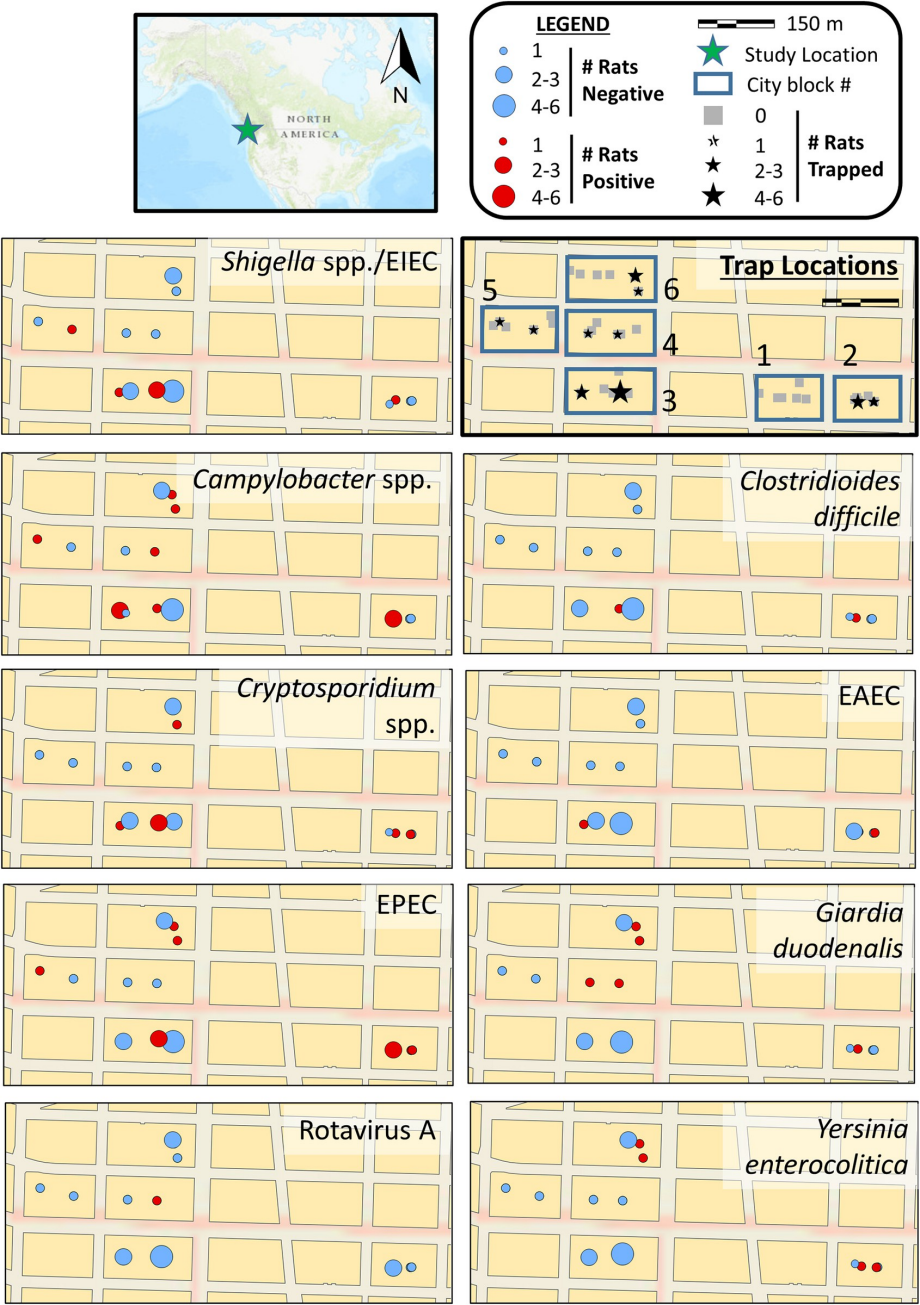
Trap locations and pathogen detection in 22 wild Norway rats (*Rattus norvegicus*) across six city blocks in the Downtown Eastside of Vancouver, British Columbia, Canada from March–April 2021. Trap location was not recorded for one rat. Pathogens detected by a multiplex PCR panel and *ipaH* PCR assay include *Shigella* spp./enteroinvasive *Escherichia coli* (EIEC), *Campylobacter* spp., *Clostridioides difficile*, *Cryptosporidium* spp., enteroaggressive *E*. *coli* (EAEC), enteropathogenic *E*. *coli* (EPEC), *Giardia duodenalis*, Rotavirus A, and *Yersinia enterocolitica* (positive = red circles; negative = blue circles). The location of Vancouver, British Columbia within North America is indicated by a green star. Maps were created using ArcGIS software (Esri, Redlands, California, USA): https://www.arcgis.com/home/item.html?id=7dc6cea0b1764a1f9af2e679f642f0f5.

Rats were trapped in the DTES neighborhood of Vancouver, Canada. We selected six city blocks for trapping that had the highest concentration of positive human *S*. *flexneri* cases (Fig 1). In each city block, we placed six traps along the alleyway for a total of 36 active traps. Lethal Snap-e rat traps (Kness Manufacturing Co., Iowa, United States) were inserted inside tempered PROTECTA EVO Express Bait Stations (Bell Laboratories, Wisconsin, United States) to prevent trap damage. Bait stations were placed along walls and fences, as rats travel along solid structures [9]. Traps were chained to immovable objects in alleyways to prevent removal and were baited with Provoke Rat Gel Bait (Bell Laboratories, Wisconsin, United States) and bacon fat. Rats were trapped from March 23 to April 9, 2021. Trapping occurred on weekdays with traps being fixed open Friday–Sunday. During active trapping, traps were checked for rats each morning and any sprung traps were reset. For each trapped rat, the date of capture and their location were recorded. After collection, carcasses were stored at -20°C for 6–21 days prior to necropsy.

Rats were thawed at 4°C and underwent a complete necropsy. We collected morphological information including species, sex, sexual maturity (perforate vagina for females; scrotal testes for males), weight (grams), body condition score based on quantity of subcutaneous and visceral fat stores (1 = poor; 2 = moderate; 3 = good), presence and number of bite wounds, and if female, whether pregnant and parous (S1 Table). Using aseptic technique, we collected each rat’s whole intestine which were stored at -80°C for 42–46 days prior to ancillary testing.

### Sample analysis

#### Multiplex PCR detection of human enteropathogens

Portions of the rat colon samples with fecal content were incised and transferred to a lysate tube filled with 0.1 mm glass beads (Biospec; Bartlesville, OK), and 2 mL of Cary Blair Media. The sample was lysed using the Qiagen Tissue Lyser for 3 minutes at 50 oscillations/s and then centrifuged for 2 minutes at 13,000 rotations per minute. 200 μL of the samples were then transferred to the FilmArray Gastrointestinal Panel (Biofire Diagnostics, Salt Lake City, UT). This assay is certified for human stool samples, and tests for the following pathogens: *Campylobacter* spp., *C*. *difficile*, *Yersinia enterocolitica*, *Cryptosporidium* spp., *Giardia duodenalis*, Rotavirus A, enteroaggressive *E*. *coli* (EAEC), enteropathogenic *E*. *coli* (EPEC), and *Shigella* spp./enteroinvasive *E*. *coli* (EIEC). Positive controls contained human stool samples with *S*. *flexneri*. The negative control consisted of sterile water added to the beads with Cary Blair media.

#### Targeted detection of *Shigella* spp. by PCR

These samples were the subject of an investigation into the relatedness of *Shigella flexneri* isolated from humans and rats within the outbreak area, and are described in detail in Ritchie *et al*. [10]. In brief, for the multiplex PCR, 25 μL of 10% SDS and 25 μL of 25 mg/mL Proteinase K were added to 500 μL of bead-lysed rat intestine sample and incubated at 65°C for 1 hr with shaking. DNA for *ipaH* and IS-PCR was extracted on the Roche MagNA Pure Compact.

To confirm *Shigella* spp./EIEC results from the multiplex assay, a region of the *ipaH* gene common to *Shigella* spp. and EIEC was amplified by PCR, based on Thiem *et al*. [11]. The primer and probe sequences were as follows: ipaHF, 5’-CCT TTT CCG CGT TCC TTG A-3’; ipaHR, 5’- CGG AAT CCG GAG GTA TTG C-3’; probe, 5’-FAM CGA CCT GTA GAT AAT GCT CCT CCG CT ABkFQ-3’. The 20 μL PCR reaction contained 10 μL of Gene Expression Master Mix (Integrated DNA Technologies), 0.2 μM primers, 0.1 μM probe, 1 mg/mL BSA (Ambion, Austin, TX) and 5 μL of sample DNA. The reaction conditions consisted of denaturation at 95°C for 3 min, followed by amplification with 60 cycles of denaturation at 97°C for 5 s, annealing at 60°C for 20 s, and extension at 72°C for 2 s. PCR was performed on the Roche Lightcycler 480 with reaction products detected at 510 nm.

As the *ipaH* assay cannot be used to differentiate between *Shigella* spp. and EIEC, two mobile element insertion sites unique to the human outbreak *S*. *flexneri* isolates were identified [10]. Hydrolysis probe PCR assays amplifying these insertion sites were designed to determine if the rat colonic samples contained these unique insertion sites, which would be indicative of relatedness between *Shigella* spp. rat and human isolates.

### Data analysis

Trap effort was calculated by number of traps multiplied by number of days active. Trap success was determined by number of trapped rats divided by trap effort and multiplied by 100, and adjusted according to Nelson and Clarke [12].

The distribution of rats carrying each pathogen was visualized spatially using ArcGIS Pro 2.8.1 (Esri, Redlands, California, USA). The Esri World Topographic Map used as a basemap is available at: https://www.arcgis.com/home/item.html?id=7dc6cea0b1764a1f9af2e679f642f0f5.

The estimated prevalence of each enteric pathogen carried by the rats, with 95% confidence intervals were determined by the Wilson Method. These values were calculated using R Statistical Software (v.4.0.3; R Development Core Team, Vienna, Austria) using the “prevalence” package and propCI function.

## Results

Twenty-two Norway rats (*Rattus norvegicus*) were collected over the study period. Animals were collected in 11/36 (31%) traps across 5/6 (83%) blocks; trap location was not recorded for one rat. Overall trap success was 0.06 over 378 trap nights. Of the study population, 19 (86%) were male and seven (37%) were sexually mature. One bite wound was present on one sexually mature male. Among sexually mature females (*n* = 2), one (50%) was parous and neither were visibly pregnant. Median weight was 71.3 g (range: 50.5–468.2 g) and median body condition score was two out of three (range: 1–3).

The multiplex PCR panel detected *Campylobacter* spp. (9/22; 41%, 95% CI [23–61]), *C*. *difficile* (3/22; 14%, 95% CI [5–33]), *Y*. *enterocolitica* (5/22; 23%, 95% CI [10–43]), *Cryptosporidium* spp. (8/22; 36%, 95% CI [20–57]), *G*. *duodenalis* (5/22; 23%, 95% CI [10–43]), Rotavirus A (1/22; 5%, 95% CI [1–22]), EAEC (2/22; 9%, 95% CI [3–28]), EPEC (10/22; 45%, 95% CI [27–65]), and *Shigella* spp./EIEC (3/22; 14%) (Table 1). Five of nine samples that underwent further testing were positive using the *ipaH* PCR assay for targeted detection of *Shigella* spp./EIEC, including the three samples previously identified by multiplex PCR assay and two additional samples. Overall, five rats were positive for *Shigella* spp./EIEC using the multiplex and *ipaH* PCR assays. Two rats trapped on blocks #2 and #3 were PCR positive for insertion sites unique to *S*. *flexneri* isolates from the human outbreak.

**Table 1:** Enteropathogens detected^a^ in 22 wild Norway rats (*Rattus norvegicus*) trapped in different city blocks, in Vancouver, Canada from March to April 2021 during an ongoing human *Shigella flexneri serotype* 2a outbreak.

Sample Identification	*Block*	*Shigella* spp./ Enteroinvasive *E*. *coli*^*b*^	*Campylobacter* spp.	*Clostridioides difficile*	*Yersinia entercolitica*	*Cryptosporidium* spp.	*Giardia duodenalis*	Rotavirus A	Enteroagressive *E*. *coli*	Enteropathogenic *E*. *coli*
V01	Unknown	- (*ipaH*^-^, IS^-^)	-	+	+	+	-	-	-	+
V02	3	- (*ipaH*^-^, IS^-^)	+	-	-	+	-	-	-	-
V03	3	-	-	-	-	+	-	-	-	+
V04	3	-	-	+	-	-	-	-	-	+
V05	3	+ (*ipaH*^+^, IS^+^)	+	-	-	-	-	-	-	-
V06	3	+ (*ipaH*^+^, IS^-^)	-	-	-	-	-	-	+	-
V07	4	-	+	-	-	-	+	+	-	-
V08	2	+ (*ipaH*^+^, IS^+^)	+	+	-	+	+	-	-	+
V09	6	-	+	-	+	+	+	-	-	+
V10	3	- (*ipaH*^-^, IS^-^)	-	-	-	+	-	-	-	-
V11	6	-	-	-	+	-	-	-	-	+
V12	5	- (*ipaH*^+^, IS^-^)	-	-	-	-	-	-	-	-
V13	4	-	-	-	-	-	+	-	-	-
V14	6	-	-	-	-	-	-	-	-	-
V15	2	-	-	-	-	-	-	-	+	+
V16	3	-	-	-	-	-	-	-	-	-
V17	2	- (*ipaH*^-^, IS^-^)	+	-	+	-	-	-	-	+
V18	2	-	-	-	+	+	-	-	-	+
V19	5	-	+	-	-	-	-	-	-	+
V20	3	-	+	-	-	-	-	-	-	-
V21	3	- (*ipaH*^+^, IS^-^)	-	-	-	+	-	-	-	-
V22	6	-	+	-	-	-	+	-	-	-
TOTAL		3 (ipaH– 5, IS– 2)	9	3	5	8	5	1	2	10
PREVALENCE % [95% CI]		23 [10–43]	41 [23–61]	14 [5–33]	23 [10–43]	36 [20–57]	23 [10–43]	5 [1–22]	9 [3–28]	45 [27–65]

^a^Pathogens with positive and negative detection using the FilmArray Gastrointestinal Panel (multiplex PCR assay) are denoted as “+” and “-“, respectively.

^b^Nine samples were tested further for *Shigella* spp./enteroinvasive *Escherichia coli* using the *ipaH* and unique insertion site PCR assays, and are listed as positive (+) or negative (-).

Rats carrying enteropathogens were dispersed across trap locations (Fig 1). The majority of rats (16/22; 73%) carried two or more enteropathogens. The median number of enteropathogens detected per rat was two (range 0–6). No enteropathogens were detected in 2/22 (9%) animals, while 4/22 (18%) had one pathogen, 10/22 (45%) had two pathogens, 3/22 (14%) had three pathogens, and one rat (5%) each had four, five, and six enteropathogens detected in total. Animals carrying *Y*. *enterocolitica* or *Cryptosporidium* spp. were most often detected to have EPEC (Table 2). Animals with *G*. *duodenalis* were frequently carrying *Campylobacter* spp.

**Table 2:** Pathogen pairings ordered from highest to lowest frequency, detected in 22 wild Norway rats (*Rattus norvegicus*) trapped within six city blocks in the Downtown Eastside neighborhood of Vancouver, Canada from March to April 2021.

Pathogen 1	Pathogen 2	Number of rats carrying both pathogens
*Yersinia enterocolitica*	Enteropathogenic *E*. *coli*	5
*Cryptosporidium* spp.	Enteropathogenic *E*. *coli*	5
*Campylobacter* spp.	*Giardia duodenalis*	4
*Campylobacter* spp.	Enteropathogenic *E*. *coli*	4
*Campylobacter* spp.	*Cryptosporidium* spp.	3
*Clostridioides difficile*	Enteropathogenic *E*. *coli*	3
*Y*. *enterocolitica*	*Cryptosporidium* spp.	3
*Shigella* spp./EIEC^a^	*Campylobacter* spp.	2
*Shigella* spp./EIEC	*Cryptosporidium* spp.	2
*Campylobacter* spp.	*Y*. *enterocolitica*	2
*C*. *difficile*	*Cryptosporidium* spp.	2
*Cryptosporidium* spp.	*G*. *duodenalis*	2
None	None	2
*Shigella* spp./EIEC	*C*. *difficile*	1
*Shigella* spp./EIEC	*G*. *duodenalis*	1
*Shigella* spp./EIEC	Enteroaggressive *E*. *coli*	1
*Shigella* spp./EIEC	Enteropathogenic *E*. *coli*	1
*Campylobacter* spp.	*C*. *difficile*	1
*Campylobacter* spp.	Rotavirus A	1
*C*. *difficile*	*Y*. *enterocolitica*	1
*C*. *difficile*	*G*. *duodenalis*	1
*Y*. *enterocolitica*	*G*. *duodenalis*	1
Enteroaggressive *E*. *coli*	Enteropathogenic *E*. *coli*	1
*Shigella* spp./EIEC	*Y*. *enterocolitica*	0
*Shigella* spp./EIEC	Rotavirus A	0
*Campylobacter* spp.	Enteroaggressive *E*.*coli*	0
*C*. *difficile*	Rotavirus A	0
*C*. *difficile*	Enteroaggressive *E*. *coli*	0
*Y*. *enterocolitica*	Rotavirus A	0
*Y*. *enterocolitica*	Enteroaggressive *E*. *coli*	0
*Cryptosporidium* spp.	Rotavirus A	0
*Cryptosporidium* spp.	Enteroaggressive *E*. *coli*	0
Rotavirus A	Enteroaggressive *E*. *coli*	0
Rotavirus A	Enteropathogenic *E*. *coli*	0

^a^Rats were designated as positive for *Shigella* spp./Enteroinvasive *E*. *coli* (EIEC) if the samples were positive using either the multiplex or *ipaH* PCR assay.

## Discussion

This study shows that urban rats can carry multiple enteropathogens, likely acquired through direct or indirect contact with people and/or human waste. Approximately a fifth of the rats were positive for *Shigella* spp./EIEC. Notably, two rat samples had unique insertion sites present in the concurrent human *Shigella flexneri* outbreak isolates, which is supportive of relatedness between rat and human strains [10]. As these rats were carrying *Shigella* spp. isolates similar to those identified during the human outbreak, it is therefore possible that rats may have played a role in the outbreak, or were somehow exposed to *S*. *flexneri* from human feces. Despite this, evidence of direct transmission of enteropathogens between humans and rats, such as *S*. *flexneri*, is still an area of uncertainty.

Carriage of *Shigella* spp. by Norway rats has not been frequently reported. A 5% prevalence of *Shigella* spp. or EIEC was identified in New York City, USA, for which species was also not differentiated (note that the specific bacterial species was not identified as most PCR assays cannot differentiate between these two organisms) [6]. Other studies in Tehran, Iran [7] and at chicken farms in North West Province, South Africa [8] identified a 1% and 3% prevalence of *Shigella* spp. in rats, respectively. These surveys were carried out without accompanying information on whether humans were experiencing clinical disease from similar bacterial isolates.

Other enteropathogens have been previously identified in rats from a variety of geographic locations, including the neighborhood in which this study took place. For example, rats collected from the DTES in 2011–12 had a 13% prevalence of *C*. *difficile* [13]. This study population was also screened for Shiga toxin-producing *E*. coli, which was detected in 1.6% of rats (specifically serotypes O145, O103, O26, and O45) [14]. *Campylobacter* spp. have been detected in Norway rats trapped on animal farms in the Netherlands (13%) [15], Sweden (9%) [16], and Finland (20%) [17]. *Cryptosporidium parvum* has been detected in rats from New York City, USA (2%) [6] and the United Kingdom (63%) [18], while *G*. *duodenalis* has been reported in rats in Barcelona, Spain (38%) [19]. Presence of *Y*. *enterocolitica* in rats has ranged from 1% in New York City, USA [6] to 20% in Sweden [20], and studies have identified Group A rotaviruses in 20% and 33% of rats in Berlin, Germany [21] and Sao Paolo, Brazil [22], respectively.

Differences in pathogen prevalence among studies may be a result of differing study methodologies, regional differences in prevalence among humans and other reservoir species, the degree of environmental contamination, as well as the nature of rat-human/animal-environmental interactions. In a study conducted in Sweden, *Y*. *enterocolitica* was exclusively detected on pig farms and not on chicken farms nor non-farm locations and rat isolates were similar to those found in pigs, which demonstrates the influence of interspecies interactions on pathogen transmission [20]. Unfortunately, contemporary data on the prevalence of enteropathogens in DTES residents is not available.

Strikingly, the majority of rats in this study carried two or more human enteropathogens, with one animal carrying six pathogens. Urban rats from New York City, USA and across three European countries (Czech Republic, Germany, and Hungary) were previously found to have diverse microbial burdens [6,23]. In New York City, rats were found to carry eight bacterial and one protozoal species capable of causing gastroenteritis in people, with an average of 1.6 bacterial species and 3.1 viruses identified per rat [6]. In our study, the most common pairings involved EPEC and *Y*. *enterocolitica* or *Cryptosporidium* spp; however, it is unknown whether detection of multiple pathogens are simply a result of exposure or if there are interactions among specific pathogens that may promote co-infection in rats.

There were several limitations of this study, including a small sample size due to low trap success, as compared to a previous study conducted in the DTES [24]. This may have been a result of daily washing of alleyways–a practice used to reduce human enteropathogen transmission in this neighborhood. This practice may have also impacted the manner in which rats interact with traps. Similarly, washing to remove human waste could reduce rat exposure to human enteropathogens, which might result in a lower prevalence among rats than might be expected without street cleaning. It is noteworthy that the study population consisted primarily of sexually immature males, which is important because age can influence pathogen profiles in rats. For example, presence of *C*. *difficile* has been associated with younger, smaller rats [13] while *L*. *interrogans* carriage has been associated with increased body weight [25]. Another limitation of our study was that the multiplex PCR assay was limited to testing for certain pathogens such as *Shigella* spp./EIEC, and did not assess for virulence factors. This has important implications to interpreting zoonotic pathogen burden in urban rats as some bacterial species within the same genus may not be pathogenic to humans. For example, rat-adapted *Cryptosporidium* spp. [26,27] and Rotavirus A genotypes [23] not known to be zoonotic, have previously been identified. In a study from Sweden, only non-zoonotic *Giardia* spp. and *Cryptosporidium* spp. were identified from rats [16]. In addition, the sensitivity and specificity of direct multiplex PCR (FilmArray) on intestinal lysate is unknown, as it is not a manufacturer-recommended sample for testing. However, the internal controls were amplified, suggesting inhibitory substances were not present after extraction.

A final limitation was that pathogens were only detected by PCR as the carcasses were frozen after capture, which likely resulted in decreased sensitivity. Other rat enteric bacterial surveys [e.g., 6,7,14] have also frozen fecal or colonic samples prior to bacterial culture or PCR and so may have had comparable sensitivities/specificities to our study. Additionally, freezing limits the ability to culture fastidious microbes such as *Shigella* spp. As opposed to culture, PCR cannot differentiate between live and non-viable microbes. The detection of live microbes would further provide evidence that rats could play a role in the harborage or transmission of human-origin pathogens. Future studies should therefore aim to provide bacterial isolates for serotyping to enhance evidence of enteropathogen transmission between rats and humans during a disease outbreak.

## Conclusions

Overall, our study highlights that multiple human enteropathogens can be detected in urban Norway rats. What remains unknown is the degree to which rats serve as a source of infection for humans. However, given that human pathogens are being transmitted from people to rats, transmission in the opposite direction is possible, particularly among people living in under-resourced neighborhoods with minimal sanitation and significant contact with rats. For this reason, these areas should be prioritized for rat management in order to mitigate potential zoonotic risks.

## Supporting information

S1 Table Trap locations and morphological information collected from Norway rats (*Rattus norvegicus*) captured in the Downtown Eastside neighborhood of Vancouver, British Columbia from March–April 2021. M = male, F = female; Y = yes, N = no, NA = not available. Body condition was scored based on amount of subcutaneous and visceral fat stores (1 = poor; 2 = moderate; 3 = good).
(DOCX)
