# Supplementary material for: Detection of multiple human enteropathogens in Norway rats (Rattus norvegicus) from an under-resourced neighborhood of Vancouver, British Columbia
Source: PLoS Negl Trop Dis. 2023 Oct 16;17(10):e0011669. doi: 10.1371/journal.pntd.0011669 (PMC10602374; doi:10.1371/journal.pntd.0011669)
Supplement: S1 Table — M = male, F = female; Y = yes, N = no, NA = not available. Body condition was scored based on amount of subcutaneous and visceral fat stores (1 = poor; 2 = moderate; 3 = good). (DOCX) [file pntd.0011669.s001.docx]

| Norway Rat Sample ID | Date Trapped (yyyy-mm-dd) | Trap Number | Latitude | Longitude | Block Number | Body Mass (g) | Sex | Mature (yes/no) | Number of Bite Wounds | Body Condition Score | Pregnant (yes/no) | Parous (yes/no) |
| --- | --- | --- | --- | --- | --- | --- | --- | --- | --- | --- | --- | --- |
| V01 | 2021-04-06 | NA | NA | NA | NA | 94.7 | M | N | 0 | 1 | NA | NA |
| V02 | 2021-03-25 | 18 | 49.2808519 | -123.1017285 | 3 | 89.4 | F | Y | 0 | 2 | N | Y |
| V03 | 2021-03-25 | 14 | 49.2808801 | -123.1007401 | 3 | 61.0 | M | Y | 1 | 1 | NA | NA |
| V04 | 2021-04-07 | 14 | 49.2808801 | -123.1007401 | 3 | 50.5 | M | Y | 0 | 2 | NA | NA |
| V05 | 2021-03-23 | 14 | 49.2808801 | -123.1007401 | 3 | 53.4 | M | N | 0 | 2 | NA | NA |
| V06 | 2021-03-23 | 18 | 49.2808519 | -123.1017285 | 3 | 87.3 | M | N | 0 | 2 | NA | NA |
| V07 | 2021-03-26 | 24 | 49.281827 | -123.1007975 | 4 | 62.6 | M | N | 0 | 2 | NA | NA |
| V08 | 2021-03-26 | 8 | 49.2807264 | -123.0946643 | 2 | 50.8 | M | N | 0 | 1 | NA | NA |
| V09 | 2021-03-31 | 31 | 49.28254215 | -123.1002707 | 6 | 77.8 | M | Y | 0 | 2 | NA | NA |
| V10 | 2021-03-31 | 14 | 49.2808801 | -123.1007401 | 3 | 73.0 | M | N | 0 | 1 | NA | NA |
| V11 | 2021-04-01 | 35 | 49.2827812 | -123.1003603 | 6 | 104.0 | M | Y | 0 | 3 | NA | NA |
| V12 | 2021-04-01 | 27 | 49.2819039 | -123.1029369 | 5 | 71.2 | M | Y | 0 | 3 | NA | NA |
| V13 | 2021-04-01 | 20 | 49.2818438 | -123.1015491 | 4 | 468.2 | M | Y | 0 | 3 | NA | NA |
| V14 | 2021-03-24 | 35 | 49.2827812 | -123.1003603 | 6 | 89.2 | M | Y | 0 | 2 | NA | NA |
| V15 | 2021-03-24 | 12 | 49.28071 | -123.0942505 | 2 | 51.9 | M | N | 0 | 2 | NA | NA |
| V16 | 2021-03-24 | 14 | 49.2808801 | -123.1007401 | 3 | 61.2 | M | N | 0 | 2 | NA | NA |
| V17 | 2021-03-24 | 8 | 49.2807264 | -123.0946643 | 2 | 107.5 | F | N | 0 | 3 | N | N |
| V18 | 2021-03-24 | 7 | 49.2807069 | -123.094279 | 2 | 60.8 | M | N | 0 | 3 | NA | NA |
| V19 | 2021-03-24 | 25 | 49.28203715 | -123.1037991 | 5 | 54.0 | M | N | 0 | 2 | NA | NA |
| V20 | 2021-03-24 | 18 | 49.2808519 | -123.1017285 | 3 | 55.3 | M | N | 0 | 2 | NA | NA |
| V21 | 2021-03-30 | 14 | 49.2808801 | -123.1007401 | 3 | 71.3 | M | N | 0 | 2 | NA | NA |
| V22 | 2021-03-30 | 35 | 49.2827812 | -123.1003603 | 6 | 164.3 | F | Y | 0 | 3 | N | N |
